# The nuclear envelopathies and human diseases

**DOI:** 10.1186/1423-0127-16-96

**Published:** 2009-10-22

**Authors:** Ya-Hui Chi, Zi-Jie Chen, Kuan-Teh Jeang

**Affiliations:** 1 Institute of Cellular and System Medicine, National Health Research Institutes, Zhunan 35053, Taiwan, Republic of China; 2 Molecular Virology Section, Laboratory of Molecular Microbiology, National Institute of Allergy and Infectious Diseases, National Institutes of Health, Bethesda, MD 20892, USA; 3 Building 4, Room 306, 9000 Rockville Pike, Bethesda, MD 20892-0460, USA

## Abstract

The nuclear envelope (NE) consists of two membrane layers that segregate the nuclear from the cytoplasmic contents. Recent progress in our understanding of nuclear-lamina associated diseases has revealed intriguing connections between the envelope components and nuclear processes. Here, we review the functions of the nuclear envelope in chromosome organization, gene expression, DNA repair and cell cycle progression, and correlate deficiencies in envelope function with human pathologies.

## Introduction

The nuclear envelope (NE) is a specialized structure composed of two lipid membranes that separate the cytoplasm from the nucleoplasm. The outer nuclear membrane (ONM), studded with ribosomes, is an extension of the rough ER while the inner nuclear membrane (INM) contacts chromatin and the nuclear lamina, a type V intermediate filament that composes the nuclear skeleton [1]. Although the NE encapsulates chromatin, it is not a uniformly closed barrier. Rather, it has several "doors" represented by the nuclear pore complexes (NPC) that allow communication between the nucleus and the cytoplasm [2].

**Figure 1:**
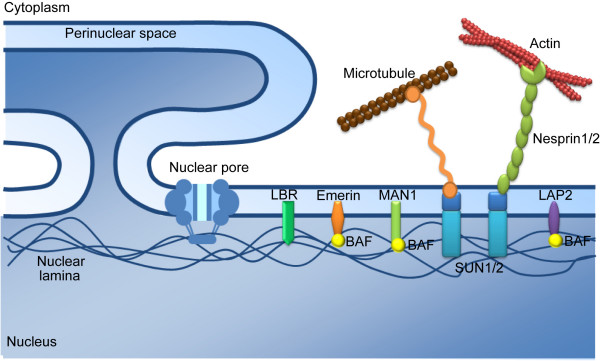
**The three-layers of NE proteins**. The nuclear pore complex (NPC) transverses the inner and outer nuclear membranes. INM proteins, including SUN1, LAP2, Emerin, MAN1 and LBR, are mostly associated with the nuclear lamina. Emerin, LAP2 and MAN1 harbor a LEM domain which interacts with BAF (barrier-to-autointegration factor), a chromatin-binding protein. The nuclear lamina forms a meshwork underlying the inner nuclear membrane.

The envelope is an anchorage structure for the nuclear architecture. A number of proteins are associated with the NE. They can be categorized into three groups according to their position in the NE (Fig. 1). The first group is the trans-nuclear membrane proteins in the nuclear pore complex (NPC). The second group is the integral nuclear membrane proteins which are embedded in the inner nuclear membrane via transmembrane domains. The functions of this group are less well characterized due to their insolubility; however, some of the better studied moieties in this group include the Lamin B receptor (LBR), the Lamin-associated polypeptides (LAPs), Emerin, MAN-1 and the Sad1-Unc84 (i.e. SUN) domain proteins. The third group of NE-associated proteins include those underlying the nuclear membrane [3]. Thus, a subset of NE-associated proteins composes the filamentous framework which constitutes nuclear structure and can regulate cellular processes. Beyond their mechanical functions, the NE proteins are involved in many nuclear activities such as DNA replication, chromatin segregation, cell cycle progression, gene transcription, and RNA processing [4]. Below, we review in brief recent perspectives on the NE proteins and how their deficiencies impact human diseases.

### NE-associated human diseases

Many human diseases are associated with NE protein defects [5]. For example, a comprehensive proteomic study identified 13 known and 67 putative INM proteins, 23 of these proteins map to chromosome regions linked to a variety of dystrophies including congenital muscular dystrophy, Charcot-Marie-Tooth diseases and myotubular myopathy [6].

A great number of NE-associated diseases (Table 1) are directly or indirectly linked to inherited mutations in Lamin A (i.e. *LMNA*), a component of the nuclear lamina [7]. Mutations in *LMNA *have been found in 11 clinically distinct tissue-specific degenerative diseases collectively termed "laminopathies", including muscular dystrophy, cardiomyopathy, lipodystrophy, and progeria (Table 1) [8]. These laminopathies can be broadly divided into two groups: first, neuromuscular disorders affecting directly the skeletal muscle, cardiac muscle and peripheral nervous system [*e.g*. Emery-Dreifuss muscular dystrophy (EDMD), dilated cardiomyopathy (DCM), Limb-girdle muscular dystrophy 1B (LGMD1B)]; second, partial lipodystrophy syndromes with or without developmental abnormalities and premature aging [*e.g*. mandibuloacral dysplasia and Hutchison-Gilford Progeria Syndrome (HGPS)].

**Table 1:** Diseases caused by mutations in nuclear envelope proteins.

Affected Gene	Disease	Protein defects	Refs
Lamin A	Hutchinson-Gilford progeria syndrome	Deleted 50 a.a. at carboxyl terminal	[63]
	Atypical Werner syndrome	A57P, R133L, L140R	[64]
	Emery-Dreifuss muscular dystrophy, type 2	missense mutations	[65]
	Emery-Dreifuss muscular dystrophy, type 3	H222Y	[66]
	Limb-gridle muscular dystrophy type 1B	missense mutations	[67]
	Dilated cardiomyopathy, type 1A	missense mutations in exons 1 or 3	[68]
	Charcot-Marie-Tooth disorder type 2B1	R298C	[69]
	Familial partial lipodystrophy, Dunnigan type	missense mutations in exons 8 and 11	[70,71]
	Mandibuloacral dysplasia	R527H, K542N, A529V, R527C/R471C	[72]
	Restrictive dermopathy	partial loss of exon 11	[73]
	Generalized lipoatrophy/lipodystrophy	R133L and T10I	[74]
Emerin	X-linked Emery-Dreifuss muscular dystrophy	S54F, P183T, P183H, Del95-99, Del236-241	[75,76]
Lamin B receptor	Pelger-Huët anomaly	defective splicing	[12]

Of the laminopathies, HGPS is one of the more severe syndromes with affected individuals having a mean life span of approximately 13 years. More than 90% of HGPS are caused by a silent mutation (GGC>GGT; G608G) which leads to a mis-spliced form of Lamin A that is deleted for 50 amino acids at its C terminus. This mutated form of lamin A is termed "Progerin" [9]. Patients expressing Progerin have aging-like features such as alopecia, sclerosis, wrinkling and arteriosclerosis at a prematurely early age [9].

Mutations in NE proteins Emerin or Lamin A/C may cause Emery-Dreifuss muscular dystrophy (EDMD; Table 1). EDMD is characterized by slowly progressive skeletal muscle wasting of the shoulder girdle and distal leg muscles, early contractures of the elbows and Achilles tendons, and cardiomyopathy with atrial ventricular block which eventually results in death [10,11].

The Lamin B receptor (LBR), a member of the sterol reductase family, is evolutionarily conserved and integral to the inner nuclear membrane. LBR targets heterochromatin and lamin proteins to the nuclear membrane. Mutations in LBR cause the Pelger-Huët anomaly (PHA), an autosomal dominant disorder characterized by abnormal nuclear shape and chromatin disorganization in blood granulocytes (Table 1). Affected individuals show hypolobulated neutrophil nuclei with rough chromatin. Presumptively homozygous individuals have ovoid neutrophil nuclei, as well as varying degrees of developmental delay, epilepsy, and skeletal abnormalities [12].

### Mouse models of nuclear envelopathies

Mouse models have been generated recently to investigate the biological relevance of different NE genes. Animals from most of these models exhibit developmental retardation and tissue-specific defects. Below, we enumerate the various mouse phenotypes that emerge from deficiencies in various NE components.

### Mouse models for nuclear lamins

#### Lamin A

Mammalian somatic cells express three types of lamins, Lamin A (*LMNA*), Lamin B (*LMNB*) and Lamin C (*LMNC*). Lamin A and Lamin C are splicing variants that are identical in their first 566 residues, but are distinct at their carboxyl-terminal ends. The expression of Lamin A/C is tissue-specific and developmentally regulated, whereas the Lamin B protein is ubiquitously expressed in all mammalian tissues [13]. In the mouse, A-type lamins do not appear until midway through embryonic development, suggesting that these proteins may be involved in regulating terminal differentiation. Mutations in *LMNA *directly affect the nuclear morphology as observed in human laminopathies [14]. Indeed, knock out of *Lmna *in mice disturbs the actin, vimentin and tubulin-based cytoskeletal filamentous networks in *Lmna*^-/- ^mouse embryonic fibroblasts [15]. Although *Lmna*^-/- ^mice are born as animals grossly indistinguishable from their wild type littermates, they soon display pathologies such as cardiomyopathies, lipodystrophies, alopecia and distinct scoliosis/kyphosis [15]. It has been reported that all *Lmna*^-/- ^mice perish by the eighth week after birth [15]. Interestingly, Fong *et al*. showed that *Lmna*^*LCO*^/^*LCO *^mice which produce Lamin C but no Lamin A or prelamin A are entirely healthy, and *Lmna*^*LCO*^/^*LCO *^cells displayed normal Emerin localization and exhibited only minimal alterations in nuclear shape and nuclear deformability [16]. On the other hand, mice carrying an autosomal recessive mutation in the Lamin A gene display HGPS-like pathological defects in bone, muscle and skin and death by 4 weeks of age [17]. Thus, prelamin A and Lamin A seem to be dispensable in mice; the symptoms in laminopathies may due to the dominant negative effect of the Lamin A mutant proteins.

#### Lamin B1

Although spontaneous mutations in B-type lamins have never been identified in humans or in experimental animals, the Lamin B1 mutant mice may provide evidence for a broad and non-redundant function of Lamin B1 in mammalian development. Lamin B1 is expressed in embryos and differentiated cells; homozygous *Lmnb1 *mutant mice live until birth, albeit with bone and lung abnormalities [18]. Fibroblasts from mutant embryos grow under standard cell-culture conditions, but display grossly misshapen nuclei, impaired differentiation, increased ploidy, and premature senescence [18].

### Mouse models for INM genes

Most of the known INM proteins are associated with the nuclear lamina [19]. Several mouse models have been established to investigate the physiological consequences of a loss of interaction between the nuclear lamina and the INM proteins.

#### SUN-domain genes

The mammalian SUN-domain proteins are inserted into the INM with their N-termini interacting with Lamin A and their C-termini connecting to the cytoplasmic architecture through direct interactions with Nesprin [20-22]. The SUN domain proteins have various functions in eukaryotic cells. The *Schizosaccharomyces pombe *SUN-domain protein *Sad1p *is a constituent of the spindle pole body (SPB) which contacts the telomere complex [23]. *Mps3p*, the single SUN-domain protein in *Saccharomyces cerevisiae*, is required for NE anchorage-dependent recombinational repair of double-stranded DNA breaks [24,25]. On the other hand, the *C. elegans *SUN-domain protein Unc-84 is required for nuclear migration and anchorage [26]. In mice, knock out of the *Sun1 *alleles does not appear to affect birth or a normal life span. However, *Sun1*^-/- ^animals are reproductively infertile [27,28]. It was noted that mouse Sun1 specifically associates with telomeres during the leptotene and diplotene stages of meiotic prophase I. Knock out of Sun1 in mice impairs telomere attachment to the nuclear envelope, resulting in persistent double-strand breaks and inefficient homologous pairing and synapsis formation in meiosis [27,28]. Moreover, *Sun1*^-/- ^spermatocytes show repressed expression of reproductive genes and have no detectable piRNA [28]. Unlike *Sun1*^-/- ^animals, mice knocked out for *Sun2 *do not present with apparent abnormalities and are reproductively normal [29]. On the other hand, mice deficient for both *Sun1 *and *Sun2 *die at birth and show destructive myonuclear positioning in the syncytial skeletal muscle cells [29]. These findings establish a non-redundant role for Sun1 in tethering telomeres to the NE and in gene expression.

#### Lamin B receptor

The human PHA disease caused by mutation in the Lamin B receptor (LBR) gene is modeled by the ichthyosis (ic) mice. Frameshift mutations in the *ic *locus (1088insCC and 1884insGGAA) within the *Lbr *gene lead to marked abnormalities in nuclear heterochromatin. Mice homozygous for deleterious alleles at the *ic *locus develop abnormalities including alopecia, variable expression of syndactyly and hydrocephalus and present blood phenotype similar to PHA [30].

#### Lap2α

Lamin-associated polypeptide 2α (Lap2α) is a chromatin-associated protein that binds A-type lamins. The Lap2α deficient mouse model shows that Lap2α is required for nucleoplasmic localization of Lamin A. Loss of Lap2α-Lamin A interaction impairs pRb-mediated regulation of progenitor cell proliferation, leading to inefficient cell-cycle arrest in dense fibroblast cultures and the hyperproliferation of epidermal and erythroid progenitor cells *in vivo *[31].

### Mouse models for NPC (nuclear pore complex)

In addition to significant changes in the nuclear shape, the Hutchinson-Gilford progeria syndrome (HGPS) is associated with prominent clustering of nuclear pores [32]. While the link between spatial distribution of nuclear pores and a premature aging phenotype remains to be elucidated, recent findings in mouse models suggest that NPCs have additional functions in chromatin organization, gene regulation and cell cycle progression which are independent of the roles they play in nuclear transport [33]. Below, we survey mouse data on the involvement of the nuclear pore proteins in protecting chromosomal integrity.

#### Nucleoporins (Nups)

The nuclear pore complex disassembles and reassembles together with the nuclear envelope during cell division. How post-mitotic nuclear envelope reformation affects interphase chromatin organization is largely unknown [1]. When disassembled, many of the nuclear pore proteins appear not to be degraded, but are recruited instead to kinetochores. These kinetochore-associated nucleoporins participate in the activity of the spindle assembly checkpoint. For example, the RNA export 1 (RAE1)-NUP98 complex is involved in RNA export in interphase. In mitosis, RAE1 binds to microtubules and is required for spindle formation [34]. The *Rae1*^+/-^*Nup98*^+/- ^compound mice show premature securin degradation, leading to precocious anaphase onset and aneuploidy [35]. In another study, double haploinsufficiency in the mitotic checkpoint genes *Bub3 *and *Rae1 *appear to be causal of several aging-associated phenotypes [36]. Mouse embryonic fibroblasts (MEFs) from *Bub3*^+/-^*Rae1*^+/- ^mice show premature senescence and accumulate high levels of p19, p53, p21, and p16 [36]. These results link the mitotic checkpoint gene with nucleoporins in regulating chromosome integrity, cellular senescence, and aging.

#### Nuclear pore associated proteins, Mad1/Mad2

The mitotic arrest deficient protein 1 (MAD1) and 2 (MAD2) are required for the faithful bipolar attachment of the mitotic spindles to kinetochores. MAD1 and MAD2 associate with NPC in interphase [37,38]. As the nuclear membrane commences breakdown in mitosis, MAD1 and MAD2 translocate to the kinetochores via a polo-kinase dependent activity [39,40]. In mice, the homozygous knock out of either *Mad1 *or *Mad2 *is embryonic lethal [41,42]. Mice that are heterozygously insufficient for either *Mad1 *(i.e. *Mad1*^+/-^)or *Mad2 *(i.e. *Mad2*^+/-^) show a higher proclivity than wild type mice in developing tumors [41,42]. In the setting of a *p53*^+/- ^background, there is some evidence that *Mad1*^+/-^*p53*^+/-^, *Mad2*^+/-^*p53*^+/-^, and *Mad1*^+/-^*Mad2*^+/-^*p53*^+/- ^mice have more frequent presentation per mouse of multiple independent primary tumors of different tissue types [43,44]. The findings suggest that simultaneous loss of the *Mad1/Mad2 *spindle assembly checkpoint (SAC), which is often targeted by oncogenic viruses [45], and the p53 checkpoint holds a worse prognosis in mice for the development of multiple independent tumors in different tissues than the singular loss of either the SAC or the p53 checkpoint.

### The influence of the NE on chromosome organization and gene expression

The eukaryotic genome is tightly compacted to fit into the confines of the nucleus. Accumulating evidence agree that the three-dimensional distribution of the chromosomes in the nucleus is nonrandom and falls into "territories" [46,47]. Thus, the transcriptional state of genes may be dictated by how they are positioned and distributed in different nuclear space [48]. Based on electron microscopic findings, the nuclear periphery was initially thought to be occupied by transcriptionally silent heterochromatin. Recent evidence, however, suggests that the association of chromatin with the NE can result in gene activation [49]. Thus, in yeast, transcriptional activation correlates with chromatin association to NPCs, and the activation of certain genes often results in their dynamic relocation to the nuclear periphery [49-51]. In some respects, it is biologically parsimonious that gene transcription occurs at the nuclear periphery because the synthesized mRNA can then be readily exported through the nuclear pores. On the other hand, genome-wide analysis in *Drosophila *cells has also revealed clusters of transcriptionally silent and hypo-acetylated genes that interact with Lamin B1 and are positioned at the nuclear periphery [52]. Using three-dimensional DNA-immuno-FISH, Reddy *et al*. demonstrated that an Emerin, Lamin A, Lamin B and LAP2 complex can result in establishing a distinctively repressive chromatin structure that silences transcription [53]. Within this complex, proteins like LAP2 recruits histone deacetylases like HDAC3, causing deacetylation of histone H4 leading to transcriptional repression [54]. In distinction with LAP2, the human SUN-domain protein 1 appears to recruit a histone acetyltransferase that acts to regulate chromatin decondensation [20]. Collectively, the findings suggest that NE and NE-associated proteins can recruit chromatin and regulatory factors that either activate or inactivate gene transcription. In instances of aberrant loss of heterochromatin regulation, this finding is often pathologically associated with prematurely aging HGPS cells [55].

### NE and DNA repair

**Figure 2:**
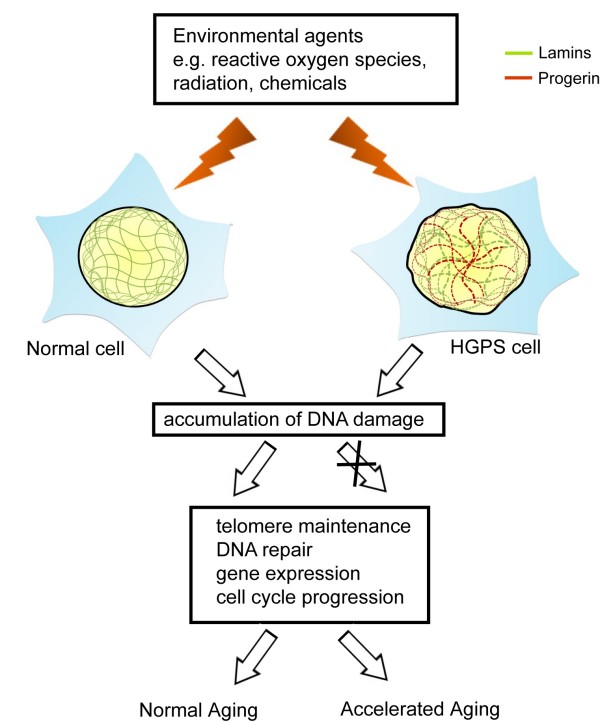
**Nuclear integrity and genome stability in normal and HGPS cells**. In normal cells, DNA damage arises from exposure to environmental fctors including reactive oxygen species, radiation or chemicals. This damage can be readily repaired by the DNA repair machinery. On the other hand, the incorporation of Progerin (brown lines) in the nuclear lamin network (green lines) alters nuclear morphology, resulting in defective nuclear processes for the maintenance of telomere length and DNA damage responses. In Progerin-expressing cells, accelerated aging ensues.

Individuals with progeroid syndromes show accelerated aging. Molecularly, aging is a process characterized by the accumulation of somatic damages and the progressive failure in the homeostasis of gene maintenance - repair whose imbalance can trigger apoptosis and senescence (Fig. 2) [56,57]. A-type lamins are necessary for normal DNA strand rejoining after exposure to irradiation [58]. Thus, HGPS fibroblasts have delayed DNA-damage checkpoint response and defective DNA repair (Fig. 2), suggesting that genomic instability may contribute to laminopathy-based premature aging [59]. Indeed, in addition to delayed DNA repair responses, telomeres in fibroblasts from HGPS patients are shorter than in age-matched controls suggesting that altered telomere dynamics and/or structure may be an additional underlying component of progeroid disease (Fig. 2) [58,60].

### The mitotic role of NE proteins

**Figure 3:**
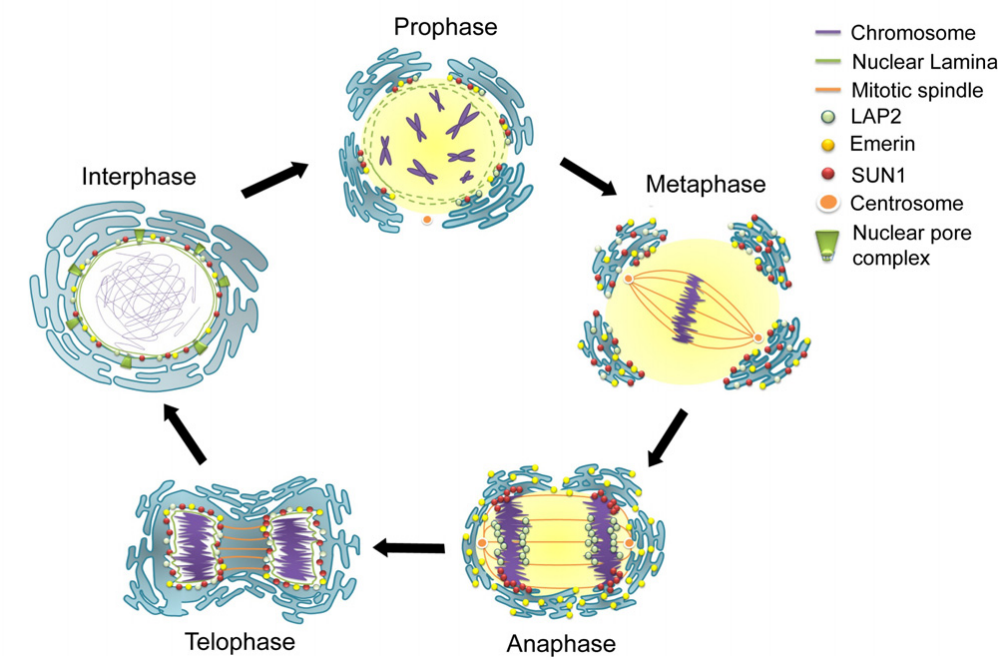
**Nuclear envelope breakdown and reassembly in mitosis**. At the end of G2, the activation of cyclin-dependent kinases, including CDK1, triggers entry into mitotic prophase. The nuclear membrane breaks down, and the NE-associate proteins either translocate to kinetochores, distribute with the fragmented ER networks, or dissolve in the cytoplasm. During NE reassembly in anaphase, SUN1 and LAP2 first appear around the condensed chromatin, though at different regions. The nuclear lamins then join the nuclear periphery in telophase. This figure illustrates the important roles played by the NE and the nuclear lamina in normal mitosis.

In higher eukaryotic cells when the NE breaks down, the nuclear lamina depolymerizes into both soluble and membrane-associate pools (Fig. 3). NPC subunits are either translocated to kinetochores or dispersed into the cytoplasm, while the nuclear membrane and INM proteins merge into the ER network. During the anaphase/telophase transition, the NE components reassemble around the condensed chromatin (Fig. 3).

At the conclusion of mitosis as the NE reforms, the NE protein complex associates with discrete regions of mitotic chromatin in a sequential manner (Fig. 3). LAP2 and a subfraction of BAF form defined complexes in chromatin core regions [20,61]. SUN1 and a subset of nucleoporins wrap the lateral margins of newly separated sister chromatids (Fig. 3). The nuclear lamina and Emerin then coalesce with SUN1 and LAP2 in telophase [20]. Thus, chromatin-interacting membrane proteins such as LBR, MAN1, LAP2, and the trans-membrane nucleoporins Ndc1 and POM121 collaborate to rapidly reestablish the nuclear compartment at the end of mitosis and facilitate NE reformation [62]. In the newly reformed nucleus, the SUN1 protein recruits a membrane associated histone acetyltransferase hALP to acetylate histones to decondense the DNA, thereby preparing the cell for transcription during the next interphase [20]. The complexity of these events underscores the importance of nuclear envelope physiology to the normal life cycle of the cell and explains why envelope defects lead to significant diseases.

### Concluding Remarks

Here, we review in brief the pleiotropic functions of NE and NE-associated proteins in normal and pathological physiology. Based on extant data, it is clear that defects in NE-associated proteins can manifest as devastating diseases such as the progeroid syndromes. What is less clear is whether the syndromes occur due to a loss of a normal function (i.e. Lamin A mutation) or a gain of an abnormal dominant negative activity (i.e. expression of a mutant Progerin protein) or both. Going forward, the study of envelopathies and laminopathies may reveal balanced contributions from both losses and gains in functions to disease development. While NE proteins were originally thought to serve only simple structural roles, much more remain to be discovered about these factors for their complex regulatory functions.

## Competing interests

The authors declare that they have no competing interests.

## Authors' contributions

YHC and KTJ wrote the manuscript. ZJC assisted with the preparation of the figures. All authors discussed the content of the writing, read and approved the final manuscript.
